# Integrated MicroRNA and mRNA Profiling in Zika Virus-Infected Neurons

**DOI:** 10.3390/v11020162

**Published:** 2019-02-16

**Authors:** Francine Azouz, Komal Arora, Keeton Krause, Vivek R. Nerurkar, Mukesh Kumar

**Affiliations:** 1 Department of Tropical Medicine, Medical Microbiology and Pharmacology, Pacific Center for Emerging Infectious Diseases Research, John A. Burns School of Medicine, University of Hawai’i at Mānoa, Honolulu, HI 96813, USA; azouzf@hawaii.edu (F.A.); krausek@hawaii.edu (K.K.); nerurkar@hawaii.edu (V.R.N.); 2 Department of Biology, College of Arts and Sciences, Georgia State University, Atlanta, GA 30303, USA; karora@gsu.edu

**Keywords:** Zika virus, flavivirus, microRNAs, neurons, neuroinflammation, anti-viral immunity

## Abstract

Zika virus (ZIKV) infections have caused a wide spectrum of neurological diseases, such as Guillain-Barré syndrome, myelitis, meningoencephalitis, and congenital microcephaly. No effective therapies currently exist for treating patients infected with ZIKV. MicroRNAs (miRNAs) are a group of small RNAs involved in the regulation of a wide variety of cellular and physiological processes. In this study, we analyzed digital miRNA and mRNA profiles in ZIKV-infected primary mouse neurons using the nCounter technology. A total of 599 miRNAs and 770 mRNAs were examined. We demonstrate that ZIKV infection causes global downregulation of miRNAs with only few upregulated miRNAs. ZIKV-modulated miRNAs including miR-155, miR-203, miR-29a, and miR-124-3p are known to play critical role in flavivirus infection, anti-viral immunity and brain injury. ZIKV infection also results in downregulation of miRNA processing enzymes. In contrast, ZIKV infection induces dramatic upregulation of anti-viral, inflammatory and apoptotic genes. Furthermore, our data demonstrate an inverse correlation between ZIKV-modulated miRNAs and target host mRNAs induced by ZIKV. Biofunctional analysis revealed that ZIKV-modulated miRNAs and mRNAs regulate the pathways related to neurological development and neuroinflammatory responses. Functional studies targeting specific miRNA are warranted to develop therapeutics for the management of ZIKV neurological disease.

## 1. Introduction

Zika virus (ZIKV) is an emerging mosquito-borne pathogen that is part of the Spondweni serocomplex of the genus Flavivirus, family *Flaviviridae*. ZIKV is closely related to other pathogens of public health importance including yellow fever virus (YFV), dengue virus (DENV), Japanese encephalitis virus (JEV), and West Nile virus (WNV). The ZIKV genome is comprised of a single-stranded, positive-sense 11-kb RNA that contains three structural and seven nonstructural genes [1,2]. ZIKV is highly neurotropic in human fetal infections and has been linked to the development of severe fetal abnormalities that include spontaneous abortion, stillbirth, hydranencephaly, microcephaly, and placental insufficiency that may cause intrauterine growth restriction [1]. An increased incidence of Guillain-Barré syndrome (GBS), neuropathy of the peripheral nervous system, has also been reported in ZIKV-infected patients [3]. No effective therapies currently exist for treating patients infected with ZIKV.

ZIKV has been shown to replicate and induce cell death in neuronal cells of fetal mice as well as in human neural progenitor cells and brain organoids, a mechanism thought to play an important role in the pathogenesis of ZIKV neurological disease [4,5,6,7]. It is known that immunocompetent adult mice are resistant to subcutaneous or intraperitoneal inoculation of ZIKV [8,9]. However, it has been demonstrated that intracerebral inoculation of ZIKV in adult immunocompetent mice results in neurological disease [10]. Additionally, several studies have reported that neonatal immunocompetent mice inoculated with ZIKV via subcutaneously or intracerebral route develop ZIKV disease, and ZIKV infection can be detected in the neurons [11,12,13]. It has also been demonstrated that ZIKV infection during the period of maximal brain growth causes microcephaly and corticospinal neuron apoptosis in wild-type mice [14]. However, to date the effect of ZIKV infection on microRNAs (miRNAs) expression in primary mouse neurons has not been examined.

miRNAs are a group of small RNAs involved in the regulation of a wide variety of cellular and physiological processes. miRNAs are considered novel diagnostic and interventional candidates due to their biochemical structure [15]. They function by directly binding to the 3’ untranslated regions (3’UTRs) of specific target mRNA, causing a block of translation or degradation of the target mRNA. miRNAs have been demonstrated to play a crucial regulatory role in neurodegenerative diseases such as Alzheimer and Parkinson [16]. miRNAs also play a critical role in the regulation of immune response; including differentiation, proliferation, cell fate determination, function of immune cells, and inflammatory mediator release as well as the intracellular signaling pathways [17,18].

miRNAs of infected cells can influence the ability of a virus to replicate or spread. It is known that endogenous miRNAs inhibit replication of a number of RNA viruses including HIV-1, Ebola virus and vesicular stomatitis virus [19,20,21,22,23]. For example, miR-28, miR-125b, miR-150, miR- 223, miR-198, and miR-382 inhibit HIV replication in CD4 T cells by directly targeting HIV RNA or by modulating cellular factors responsible for its replication [24]. Furthermore, miR-122 supports HCV replication by enhancing colony formation efficiency of HCV [25], whereas miR-196 and miR-296 substantially attenuate virus replication through type I interferon (IFN)-associated pathways in liver cells [26]. Over-expression of miRNA-30e, let-7c, and miRNA-126-5p inhibits DENV replication [27,28,29]. Cellular miR-532-5p inhibits WNV replication via suppression of host genes SESTD1 and TAB3 required for virus replication [30]. miRNA HS_154 contributes to WNV-mediated apoptosis in vitro in the human neuronal cell line, SK-N-MC [31]. Moreover, incorporation of a target sequence for cellular microRNAs expressed in the central nervous system (CNS) into the flavivirus genome alters the neurovirulence of the virus and prevents the development of lethal encephalitis in mice [32].

We have previously demonstrated the role of cellular miRNAs in the pathogenesis of WNV encephalitis [33,34]. In this study, we analyzed miRNA and mRNA profiles in ZIKV-infected neurons using the nCounter system. Unlike other platforms (such as microarray and next generation sequencing), the nCounter platform enables high throughput, sensitive, quantitative, and reproducible gene expression analysis without the need of enzymatic target amplification. This technology utilizes 100 nucleotide molecular bar codes which measure gene quantities without an amplification step [35]. To our knowledge, this is the first study to evaluate the modulation of miRNAs following ZIKV infection using relevant cells, primary neurons.

## 2. Materials and Methods

### 2.1. Neuronal Cultures

Mouse cortical neuron cultures were prepared from one-day old pups of either gender (approximately equivalent numbers) obtained from established colonies of wild-type C57BL/6J mice as described previously [36]. The neurons were plated onto poly-D-lysine-coated 6-well or 24-well plates in serum Neurobasal A medium. The cultures were maintained in serum-free Neurobasal A medium supplemented with B27 for seven days prior to infection [36,37]. This study was carried out in accordance with the recommendations of the National Institutes of Health and the Institutional Animal Care and Use Committee (IACUC). The protocol was approved by the University of Hawaii IACUC (protocol number 17-2721) and Georgia State University (protocol number A19005).

### 2.2. ZIKV Infection and Plaque Assay

In this study, we used a low cell-culture-passaged and sequence-verified ZIKV strain, PRVABC59 (BEI Resources, NR-50240). Virus strain was amplified once in Vero E6 cells and had titers of 5 × 10^6^ plaque-forming units (PFU)/mL. Cells were infected with ZIKV or PBS (Mock) at multiplicity of infection (MOI)-1 and supernatants and cell lysates were harvested at 12, 24, 48, and 72 h after infection [38]. ZIKV titers were measured in cell supernatants using plaque assay [38,39]. Experiments were repeated to obtain four biological replicates of mock- and ZIKV-infected neurons at each time point for the NanoString analysis (*n* = 4 per group per time point).

### 2.3. Indirect-Immunofluorescence Microscopy

Neuronal cell monolayers were grown on coverslips in 24-well plates and infected with ZIKV or PBS at MOI-1. Cells were fixed in 4% paraformaldehyde (PFA) and immunostained using mouse anti-dsRNA (1:1000) antibody followed by secondary antibody conjugated with Alexa Fluor 555 (Millipore, Burlington, MA, USA) as described previously [40].

### 2.4. NanoString nCounter^®^ Gene Expression

Total RNA was isolated using miRNeasy Mini Kit (Qiagen, Hilden, Germany) as described previously [33]. Genomic DNA contamination was eliminated by digesting the RNA with RNase-free DNase (Ambion, Cambridge, MA, USA). RNA was quantitated using Nanodrop (Thermo Scientific, Waltham, MA, USA), and the 28S/18S RNA ratios of all RNA samples were between 1.8 and 2.0. RNA quality was analyzed using the Bioanalyzer [33,41]. For miRNA analysis, we used the nCounter^®^ Mouse miRNA Expression Panel (NanoString, Seattle, WA, USA, Cat: CSO-MMIR15-12). Raw data was normalized using the geometric mean values of the top 100 expressed miRNA in each sample using the nSolver Analysis Software (NanoString), according to the manufacturer’s guidelines. For mRNA analysis, we utilized the nCounter^®^ Mouse PanCancer Immune Profiling Panel to count 770 immune-related genes (NanoString, Cat: XT-CSO-MIP1-12). Raw data was normalized with a set of housekeeping genes and analyzed using the nSolver Analysis Software (NanoString), according to the manufacturer’s guidelines.

### 2.5. qRT-PCR

Total RNA was isolated using miRNeasy Mini Kit, and cDNA prepared using a miScript II RT Kit (Qiagen) [33,34]. qRT-PCR was performed using specific miRNA primer (Qiagen), and the miScript SYBR green PCR kit (containing Universal reverse primer) [33]. For mRNA analysis, cDNA was prepared using iScript™ cDNA Synthesis Kit (Bio-Rad, Hercules, CA, USA), and qRT-PCR was conducted as described previously [41,42]. The primer sequences used for qRT-PCR are listed in Table 1.

### 2.6. Measurement of Cytokines and Chemokines

The levels of cytokines and chemokines were measured in the cell supernatants by multiplex immunoassay using MILLIPLEX MAP mouse Cytokine/Chemokine magnetic panel as per manufacturer’s instructions (Millipore) [43,44].

### 2.7. Ingenuity Pathways Analysis (IPA)

Target prediction and pathway analysis were conducted using IPA (Ingenuity Systems Inc., Redwood City, CA, USA) as described previously [33,34,41]. Fisher’s exact test, using IPA, was used to calculate the cut-off point of significance. *p* < 0.05 is considered significant. We also conducted correlation pairing with mRNA expression data and significantly modulated miRNAs using IPA. For multiplex immunoassay analysis, unpaired Student’s *t*-test using Graph Pad was used to calculate *p* values.

## 3. Results

### 3.1. ZIKV Can Infect Primary Mouse Cortical Neurons

**Figure 1:**
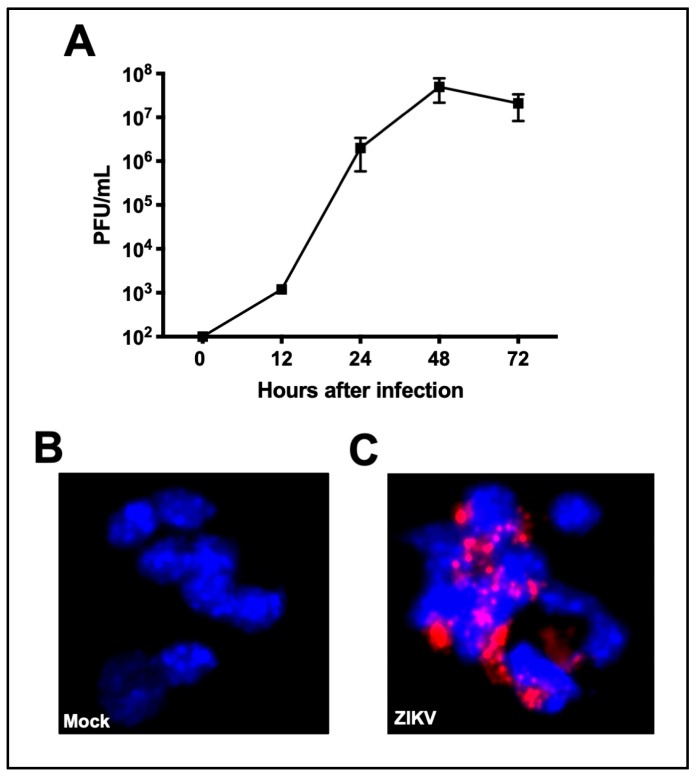
Zika virus (ZIKV) infection of the primary mouse neurons. Mouse cortical neuron cultures were prepared from one-day old pups. Neurons were infected with ZIKV (PRVABC59 strain) or PBS (Mock) at multiplicity of infection (MOI)-1. (**A**) ZIKV titers in culture supernatant were determined by plaque assay. Viral titers are expressed as plaque forming units (PFU)/mL of supernatant. Data represents the mean ± SEM. Neurons grown and fixed on coverslips at 48 h after infection were stained with anti-dsRNA antibody (red) and counterstained with DAPI (blue). (**B**) Mock-infected cells. 20× magnification. (**C**) ZIKV-infected cells demonstrate robust virus staining in the cytoplasm. 20× magnification.

ZIKV infection of neuronal cells plays an important role in the pathogenesis of ZIKV neurological disease. Therefore, we used primary neurons for our experiments. We first determined ZIKV infection and replication kinetics in primary mouse cortical neurons by plaque assay. Mouse cortical neuron cultures were infected with ZIKV (PRVABC59 strain) or PBS (Mock) at MOI-1 and supernatants were collected at 12, 24, 48, and 72 h after infection. High ZIKV replication was observed as early as 12 h after infection. Viral titers peaked at 48 h (log 7–8 PFU/mL) followed by a slight decline at 72 h after infection (Figure 1A). Immunofluorescence staining of ZIKV-infected neurons demonstrated robust dsRNA staining in the cytoplasm. Based on a total of 5,000 cells counted in 10 independent fields, dsRNA was detected in approximately 60% of cells at 48 h after infection (Figure 1B,C).

### 3.2. ZIKV Infection Modulates Cellular miRNA Expression

**Figure 2:**
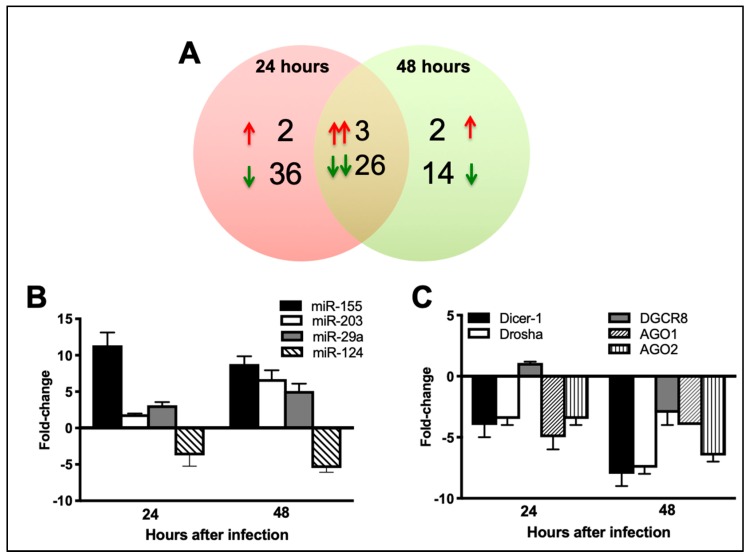
ZIKV infection of the primary mouse neurons causes changes in cellular miRNA expression. Neurons were infected with ZIKV (PRVABC59 strain) or PBS (Mock) at MOI-1. (**A**) Venn diagram showing the number of differentially expressed miRNAs at 24 and 48 h after infection. Sets of upregulated miRNAs are represented by upward red arrows and sets of downregulated miRNAs are represented by downward green arrows. Pairs of arrows in the intersection refer to the number of miRNAs upregulated (double red arrows) or down regulated (double green arrows) at both 24 and 48 h after infection. (**B**) qRT-PCR was conducted on RNA extracted from mock and ZIKV-infected neurons to determine fold-change in miR-155, miR-203, miR-29a, and miR-124-3p expression. Changes in the levels of each miRNA were first normalized to the snoRNA and then the fold-change in ZIKV-infected cells was calculated in comparison to corresponding mock-infected cells. Data represents the mean ± SEM. (**C**) qRT-PCR was conducted on RNA extracted from mock and ZIKV-infected neurons to determine fold-change in Dicer-1, Drosha, DGCR8, AGO1, and AGO2 expression. Changes in the levels of each mRNA were first normalized to the β-actin and then the fold-change in ZIKV-infected cells was calculated in comparison to corresponding mock-infected cells. Data represents the mean ± SEM.

Neurons were infected with ZIKV (PRVABC59 strain) or PBS (Mock) at MOI-1 and cell lysates were harvested at 24 and 48 h after infection. We used nCounter^®^ Technology (NanoString) to evaluate the global miRNA expression profiles in the mock-and ZIKV-infected neurons at 24 and 48 h after infection (*n* = 4 per group per time point). Among 599 miRNAs present on the array, 67 and 45 miRNAs were significantly modulated at 24 and 48 h, respectively. miRNAs that were altered at least 2-fold were considered significant. While 62 miRNAs were significantly downregulated (between 2- to 4-fold), only five miRNAs were upregulated (between 2 to 18-fold) in ZIKV-infected neurons when compared to mock-infected neurons at 24 h (Table 2 and Table 3). Similarly, 40 miRNAs were significantly downregulated (between 2- to 6-fold), and only five were upregulated (between 2 to 10-fold) at 48 h (Table 2 and Table 4). As depicted in the Venn diagram (Figure 2A), three upregulated and 26 downregulated miRNAs were common in both 24 and 48 h-infected neurons. Among upregulated miRNAs; miR-155, miR-29a, and miR-29b were induced at both 24 and 48 h. miR-3471 and miR-2145 were upregulated only at 24 h, and miR-203 and miR-1902 were upregulated only at 48 h. The miRNAs with the highest induction during the course of infection were miR-155 (18.2-fold), miR-3471 (4.9-fold), and miR-203 (4.6-fold) (Table 2). Among downregulated miRNAs, miR-124-3p was the highest downregulated miRNA at both 24 (4-fold) and 48 (6.6-fold) h. Other miRNAs with high downregulation were miR-883a-3p (3.9-fold) and miR-2137 (2.9-fold). We also conducted qRT-PCR to confirm the expression changes of a selected number of differentially expressed miRNAs. Similar to the NanoString data, miR-155, miR-29a, and miR-203 were significantly upregulated, and miR-124-3p was downregulated in ZIKV-infected neurons (Figure 2B)**.**

### 3.3. ZIKV Infection Results in Downregulation of miRNA Processing Enzymes

Flaviviruses have been shown to induce downregulation in the expression of cellular miRNAs by targeting miRNA processing enzymes [45,46,47,48]. Our data also demonstrated a trend toward decrease in miRNA expression in ZIKA-infected cells. Therefore, we next evaluated the expression of miRNA processing enzymes in ZIKV-infected neurons. mRNA expression levels of Dicer-1, Drosha, AGO1, and AGO2 were downregulated in ZIKV-infected neurons as compared to mock-infected neurons at both 24 and 48 h (Figure 2C). mRNA expression of DGCR8 increased slightly at 24 h followed by a decrease in the expression at 48 h.

### 3.4. Functional Analysis of ZIKV-Modulated miRNAs and Their Predicted Targets

Biofunctional analysis of ZIKV-modulated miRNAs and their targets revealed organismal injury and abnormalities, immunological disease, inflammatory response, neurological disease, and nervous system development and function as the top pathways in signaling pathways category (Table 5). Since ZIKV infection is associated with a wide spectrum of neurological and immunological diseases, these miRNAs may play an important role in the development of brain abnormalities following ZIKV infection.

### 3.5. ZIKV Infection Induces Dramatic Upregulation of Anti-Viral, Inflammatory, and Apoptotic Genes in Neurons

**Figure 3:**
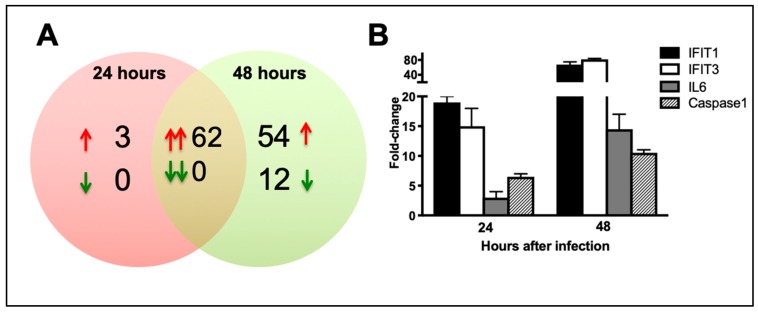
ZIKV infection of the primary mouse neurons causes changes in cellular mRNA expression. Neurons were infected with ZIKV (PRVABC59 strain) or PBS (Mock) at MOI-1. (**A**) Venn diagram showing the number of differentially expressed mRNAs at 24 and 48 h after infection. Sets of upregulated mRNAs are represented by upward red arrows and sets of downregulated mRNAs are represented by downward green arrows. Pairs of arrows in the intersection refer to the number of mRNAs upregulated (double red arrows) or down regulated (double green arrows) at both 24 and 48 h after infection. (**B**) qRT-PCR was conducted on RNA extracted from mock and ZIKV-infected neurons to determine fold-change in IFIT1, IFIT3, IL6, and Caspase1 expression. Changes in the levels of each mRNA were first normalized to the β-actin and then the fold-change in ZIKV-infected cells was calculated in comparison to corresponding mock-infected cells. Data represents the mean ± SEM.

We next analyzed the mRNA expression of key anti-viral, inflammatory, and apoptotic genes in the ZIKV-infected neurons to examine whether differentially expressed miRNAs could regulate their target mRNAs. Mouse cortical neuron cultures were infected with ZIKV (PRVABC59 strain) or PBS (Mock) at MOI-1 and cell lysates were harvested at 24 h and 48 h after infection. We utilized the nCounter^®^ Mouse PanCancer Immune Profiling Panel to count 770 immune-related genes. mRNA expression profiles for ZIKV-infected neurons were compared with mock-infected neurons. Only mRNAs that were altered at least 2-fold were considered significant. Figure 3A demonstrates total numbers of up- and downregulated differentially expressed mRNAs in ZIKV-infected neurons at 24 and 48 h. The number of differentially expressed mRNAs was higher at 48 h after infection, which correlates with significantly higher viral load observed at 48 h as compared to 24 h (Figure 1A). At 24 h, 65 mRNAs were upregulated with fold change values ranging from 2.0 to 26 (Table 6). mRNAs were not downregulated at 24 h. At 48 h, 116 mRNAs were upregulated with fold change values ranging from 2.0 to 244 (Table 6 and Table 7) and 12 mRNAs were downregulated (Table 8). 62 upregulated mRNAs were common in both 24 and 48 h- infected neurons (Table 6). MMP9, SOCS3 and IFI27 were upregulated only at 24 h. 54 mRNAs were upregulated only at 48 h (Table 7).

**Figure 4:**
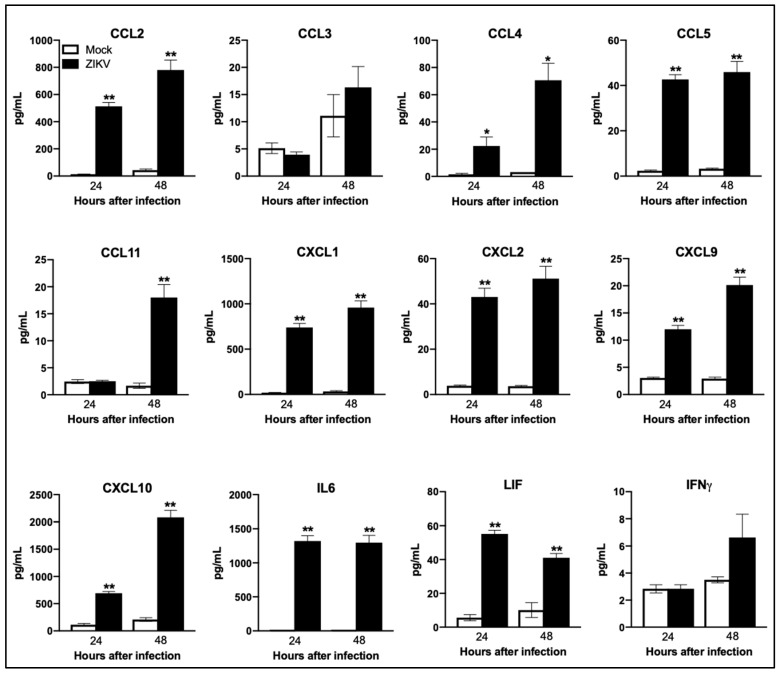
Enhanced production of cytokines and chemokines in ZIKV-infected neurons. Mouse cortical neuron cultures were infected with ZIKV (PRVABC59 strain) or PBS (Mock) at MOI-1 and supernatants were collected at 24 and 48 h after infection. Levels of chemokines and cytokines as noted in the figure were measured in cell supernatants using multiplex immunoassay and are expressed as the mean concentration (pg/mL) ± SEM. *p < 0.05, **p < 0.001.

Genes associated with virus sensing and type 1 IFN signaling were the most upregulated genes after ZIKV infection. ZIKV infection also induced a strong upregulation of multiple cytokines and chemokines in the neurons. Most of the chemokines and cytokines were significantly upregulated after ZIKV infection, including CXCL10, CCL5, CCL7, CCL2, CXCL1, CCL12, CXCL9, CXCL2, CCL4, CXCL11, CXCL13, CCL11, CXCL5, IL6, IL7, PTGS2, and LIF. Several genes involved in cell death (CASP1, TNFSF10, RIPK2, and BID) were also activated upon ZIKV infection (Table 6 and Table 7). We also validated the expression of selected upregulated mRNAs using qRT-PCR. Similar to the NanoString data, IFIT1, IFIT3, IL6, and Caspase1 were significantly upregulated in the ZIKV-infected neurons as compared to mock-infected neurons at both 24 and 48 h (Figure 3B). To examine that this increase in mRNA expression also lead to increased protein levels, we measured protein levels of key chemokines and cytokines in the cell culture supernatants using multiplex immunoassay. Similar to the mRNA expression, protein levels of the key chemokines such as CCL2, CCL4, CCL5, CCL11, CXCL1, CXCL2, CXCL9, and CXCL10, and cytokines such as IL6 and LIF were significantly increased in ZIKV-infected neurons as compared to mock-infected neurons (Figure 4). 

### 3.6. Functional Analysis of ZIKV-Modulated mRNAs

**Figure 5:**
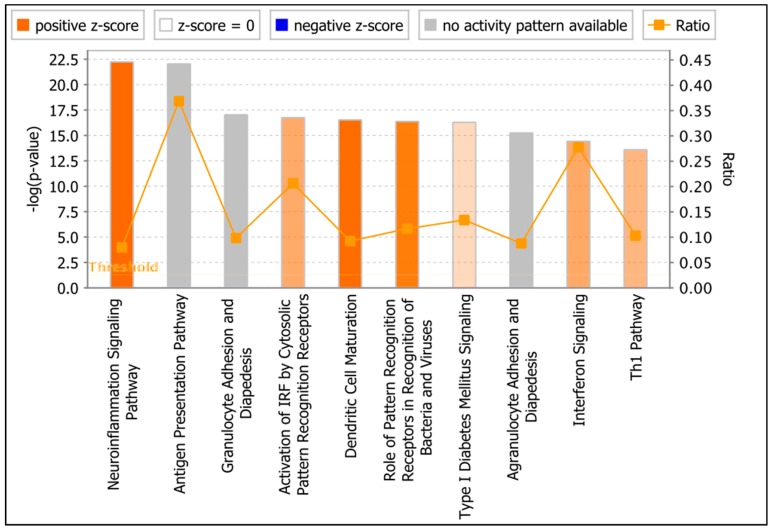
Core functional pathway analysis of ZIKV-modulated mRNAs using IPA. Top canonical signaling pathways regulated by significantly modulated mRNAs. Threshold bar indicates cut-off point of significance *p* < 0.05, using Fisher’s exact test. Range of activation z-score is also depicted in the figure. The color of the bars indicates predicted pathway activation based on z-score (orange = activation; blue = inhibition; gray = no prediction can be made; white = z-score close to 0). Orange line represents the ratio = number of genes in dataset/total number of genes that compose that pathway.

To investigate the biological interactions of differentially expressed mRNAs and identify important functional networks, significantly modulated mRNAs were imported into the IPA tool. The highest activated networks (high z-score) were identified using IPA. Figure 5 depicts the top 10 activated canonical pathways after ZIKV infection. The topmost activated canonical pathway after ZIKV infection was ‘Neuroinflammation Signaling’. In addition, key players in inflammation and innate immunity, such as ‘Activation of IRFs by Cytosolic Pattern Recognition Receptors (PRR)’, ‘Role of PRR in Recognition of Viruses and Bacteria’, and ‘IFN Signaling’ were also highly activated (positive z-score). To further understand the role of these differentially activated canonical pathways, we generated the network maps of ‘PRR in Recognition of Viruses and Bacteria’ and ‘IFN Signaling’.

#### 3.6.1. Pattern Recognition Receptors

**Figure 6:**
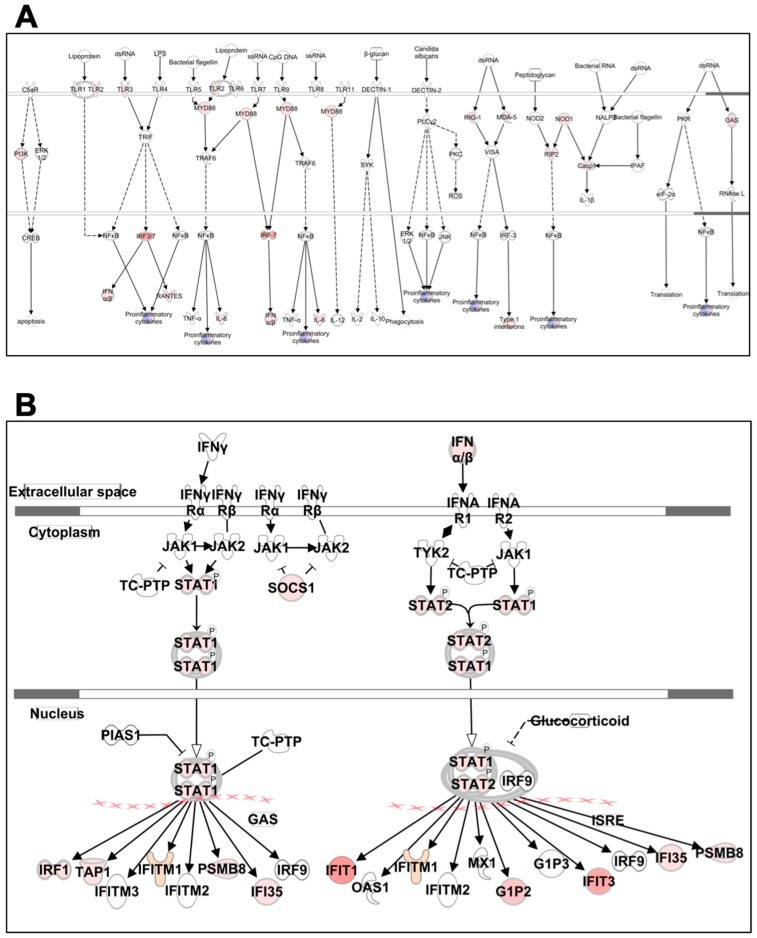
Pathway analysis for PRR and IFN signaling. Genes associated with (**A**) PRR and (**B**) IFN signaling activated by ZIKV infection are shown. Differentially expressed mRNAs are highlighted in color. Color intensity indicates the degree of upregulation (red) relative to the mock-infected neurons. Solid lines represent direct interactions and dashed lines indirect interactions. Shading intensity indicates the degree that each mRNA was upregulated.

Our data demonstrate that ZIKV infection induces the expression of all three major PRR; retinoic-acid-inducible gene-I (RIG-I)-like receptors (RLR), toll-like receptors (TLR), and the nucleotide oligomerization domain (Nod)-like receptors (NLR) (Figure 6A). The RLR are a family of cytosolic RNA helicase proteins comprised of three members: RIG-I, myeloma differentiation antigen 5 (MDA5), and LGP2 [49]. Both RIG-I and MDA5 were upregulated after ZIKV infection. The importance of the RLR signaling pathway in protection against flaviviruses has been validated by several studies in vivo [49,50,51]. The TLR family is composed of more than 10 members, with each acting as a sensor of conserved microbial component, that drive the induction of immune response [52]. Our data show that ZIKV infection induces the expression of TLR2, TLR3, and adaptor molecule MYD88. NLR are soluble or cytosolic receptors in the mammalian cell cytoplasm [53]. Activation of NOD1 and NLRC5 was observed following ZIKV infection. In addition to RLR, TLR, and NLR; PKR and OAS are classes of IFN-inducible PRR that can recognize dsRNA and restrict a number of viruses. Our data demonstrate the activation of OAS after infection with ZIKV. Furthermore, we observed increased levels of type 1 interferons and several pro-inflammatory mediators after ZIKV infection in neurons, which correlate with PRR activation in these cells.

#### 3.6.2. IFN Signaling

ZIKV infection induced strong upregulation of genes associated with IFN signaling such as IFNα, IFNβ, STAT1, and STAT2 (Figure 6B). Interferon-stimulated genes (ISG) such as MX2, IFIT1, IFIT3, IFITM1, IFI35, and RSAD2 (Viperin) were significantly upregulated after ZIKV infection. The IFN response is central to the innate defense mechanisms of the host against flavivirus infection. The paracrine and autocrine secretion of IFN creates an anti-viral state by inducing several genes including ISG [49,54].

### 3.7. Network Analysis of Expression of miRNAs and mRNAs From ZIKV-Infected Neurons

**Figure 7:**
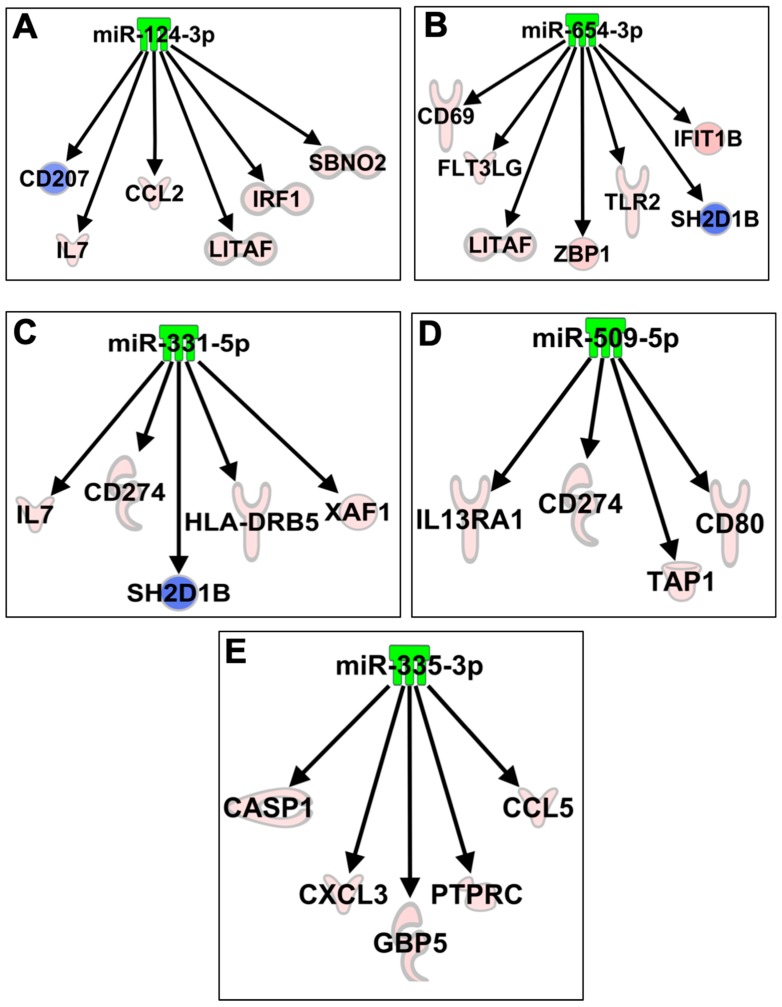
Networks of the interactions of the miRNA target genes. IPA tool was used to generate the miRNA-mRNA interaction network of (**A**) miR-124-3p, (**B**) miR-654-3p, (**C**) miR-331-5p, (**D**) miR-509-5p, and (**E**) miR-335-3p and mRNAs significantly modulated in neurons after ZIKV infection. Red (increased expression) and green (decreased expression).

We next sought to determine whether mRNA expression changes might be influenced by the differential expression of cellular miRNAs during ZIKV infection. To analyze the direct and indirect miRNA–mRNA interactions, we conducted IPA expression pairing analysis with mRNA expression data and significantly modulated miRNAs. Several miRNAs were found to directly or indirectly target multiple mRNAs analyzed in our study and demonstrated an inverse correlation with mRNA expression induced by ZIKV. These targets are predicted by TargetScan. miR-124-3p was the highest downregulated miRNA. Our data demonstrated increased expression of all the predicted targets of miR-124-3p including IL7, CCL2, LITAF, IRF1, and SBNO2 in ZIKV-infected neurons (Figure 7A). Other known targets of miR-124 includes SOCS5, TLR6, STAT3, TNF, and NF-kB [55]. Similarly, miR-654-3p was downregulated and its predicted targets—CD69, FLT3LG, IFIT1, TLR2, ZBP1, LITAF—were upregulated in ZIKV-infected neurons (Figure 7B). These targets involve genes belonging to anti-viral and inflammatory response signaling pathways. Furthermore, downregulation of miR-331-5p and miR-509-5p was inversely correlated to their predicted targets IL7, CD274, HLA-DRB5, XAF1, IL13RA1, TAP1, and CD80 (Figure 7C,D). Our data further indicate that miR-335-3p may play an important role in regulating inflammatory and apoptotic genes in the neurons following ZIKV infection. miR-335-3p targets Caspase1, CCL5, CXCL3, PTPRC, and GBP5. These genes play significant roles in mediating inflammation and cell death (Figure 7E). We also show an increase in the protein levels of the target genes such as CCL2, CCL5, and CXCL10 (Figure 4). It is interesting to see the correlation between ZIKV-modulated miRNAs and target genes at both mRNA and protein level, which is consistent with the miRNA–mRNA–protein triad and demonstrate the functional importance of our results.

## 4. Discussion

In this study, we used nCounter technology to identify miRNAs and mRNAs modulated by ZIKV infection in neurons. Our data demonstrate that ZIKV infection causes global downregulation of miRNAs with only few upregulated miRNAs. ZIKV infection also results in downregulation of miRNA processing enzymes. Upregulated miRNAs including miR-155, miR-203, and miR-29a have been previously shown to play critical role in flavivirus infection and anti-viral immunity [22,56,57,58,59,60]. ZIKV infection in the neurons significantly induced the expression of anti-viral, inflammatory and apoptotic genes. Biofunctional analysis revealed that ZIKV-modulated miRNAs and their target genes regulate the pathways related to neurological development and neuroinflammatory responses. Furthermore, our data show an inverse correlation between ZIKV-modulated miRNAs and target host immune mRNAs induced by ZIKV infection.

Flaviviruses have been shown to induce downregulation in the expression of cellular miRNAs by targeting miRNA processing enzymes [33,45,46,47,48,61]. It has been reported that depletion of Dicer and Drosha by siRNA-mediated silencing results in increase in flavivirus replication [46,47,61]. We also observed a trend towards decrease in miRNAs expression in ZIKA-infected cells. Similar to our data, ZIKV infection in human astrocytes induces global downregulation of miRNAs [62]. It has been demonstrated that ZIKV infection in *Aedes aegypti* mosquitoes downregulates expression of host miRNAs [63]. In this study, we also show that ZIKV infection results in downregulation of miRNA processing enzymes. Downregulation of genes involved in miRNA processing enzymes including Dicer-1 was also reported in ZIKV-infected astrocytes [62]. Similarly, ZIKV infection in HepG2 cells led to a decrease in DGCR8, Ago1, and Ago3 expression [64]. It is known that non-coding, subgenomic RNA (sfRNA) of WNV and DENV can interfere with miRNA biogenesis pathway. WNV sfRNA is processed by Dicer and suppresses RNAi [33,45,46,47,48,61]. Future experiments are warranted to elucidate whether this sfRNA function holds for all flaviviruses including ZIKV. Since miRNAs negatively regulate mRNA expression, decrease in cellular miRNA expression results in increase in apoptotic and inflammatory genes associated with flavivirus infection [27,30,31,33,41]. Our data also demonstrate that ZIKV infection is associated with dramatic upregulation of several anti-viral, apoptotic and inflammatory genes.

We observed significant upregulation of miR-155, miR-203, miR-29a, and miR-29b in ZIKV-infected neurons. These miRNAs have been shown to play an important role in viral infection [19,20,21,65,66]. miR-155 is multifunctional and widely reported to modulate different stages of innate immune response during inflammation and infection [21,22,56]. miR-155 not only modulates TLR-mediated innate immune response, but also targets complement regulatory proteins and facilitate complement activation [56,57]. This phenomenon is critical to eliminate the virus from infected cells. Several published studies have demonstrated the essential role of miR-155 in viral infections caused by Epstein–Barr, Borna disease, and reticuloendotheliosis viruses [19,20,56,65]. For example, overexpression of miR-155 significantly suppressed human HIV infection in activated macrophages [21]. Similarly, miR-155 suppresses JEV replication in microglial cells and regulates JEV-induced inflammatory response in mice brain [22,67]. Studies have shown that miR-203 is also involved in regulating the anti-viral immune response [59,60]. It has been demonstrated that miR-29b regulates JEV-induced neuroinflammation [58]. Since these miRNAs are known to have a role in anti-viral immunity, upregulation of these miRNAs might play a role in controlling ZIKV infection and associated neurotoxicity. A recent study identified upregulation of miR-30e-3p, miR-30e-5p, and miR-17-5p in ZIKV infection of human astrocytes [62]. However, we did not observe upregulation of these miRNAs in ZIKV-infected mouse neurons. This could be due to the difference in the neural cell type and species studied.

In our study, miRNAs with the highest downregulation were miR-124-3p, miR-883a-5p and miR-2137. miR-124 is the most abundant miRNA in the brain and affects a broad spectrum of biological functions in the CNS [55,68,69,70]. miR-124 has been reported to participate in chronic stress, neurodegeneration, alcohol/cocaine neuroadaptation, synapse morphology, neurotransmission, long-term potentiation, neurodevelopment, myeloid cell function, and hematopoiesis. In mammalian neurons, miR-124 suppresses the levels of many non-neural genes, which contributes to the acquisition and maintenance of neuronal identity [69]. Furthermore, when miR-124 is aberrantly expressed, it contributes to pathological conditions involving the CNS [68,69,70]. It has also been shown to be useful as a diagnostic and prognostic indicator of CNS disorders, such as brain tumors and stroke [68]. miR-883a has been demonstrated to be involved in inflammatory pathways, and target genes belonging to the TLR signaling pathway as well as the VEGF and chemokine signaling pathways [71]. miR-2137 is involved in the pathogenesis of traumatic brain injury [72]. IPA analysis also revealed that individual miRNA including miR-124a-3p, miR-654-3p, miR-331-5p, miR-335-3p, and miR-509-5p can modulate multiple upstream regulatory genes. There was an inverse correlation between these miRNAs and target host immune mRNAs induced by ZIKV infection. Since these miRNAs are known to have a role in brain injury and inflammatory responses, downregulation of these miRNAs following ZIKV infection possibly resulted in upregulation of neuroinflammatory and apoptotic genes. However, further experiments are required to validate their biological functions in ZIKV infection.

This study has few limitations. First, we only analyzed the mRNA expression of genes primarily involved in immune response, which introduce a bias in the analysis towards anti-viral response. Second, additional experimental studies are warranted to validate the interplay between miRNA, mRNA, and ZIKV replication, which includes knockdown of individual miRNA and its effect on the expression levels of its target mRNAs and virus titers.

In conclusion, this is the first study to evaluate the modulation of miRNAs following ZIKV infection in neurons. ZIKV-modulated miRNAs in neurons are also known to play a role in the pathogenesis of other flaviviruses and anti-viral immune response. In this study, the utility of the nCounter system enabling rapid miRNA and mRNA expression analysis was also demonstrated. Collectively, these data suggest that miRNAs regulate downstream gene expression, important in ZIKV disease pathogenesis, and can be targeted in the future to develop therapeutics for the management of ZIKV neurological disease.

## Figures and Tables

**Table 1:** Primer sequences used for qRT-PCR.

Gene (Accession No.)	Primer Sequence (5’‒3’)
IFIT1 (NM_008331)	
Forward	GTTGTTGTTGTTGTTCGT
Reverse	CAGCAGGAATCAGTTGTG
IL6 (NM_031168)	
Forward	ATCCAGTTGCCTTCTTGGGACTGA
Reverse	TAAGCCTCCGACTTGTGAAGTGGT
IFIT3 (NM_010501)	
Forward	GTCCTCTCTACTCTTTGG
Reverse	CATCCTCTGTCTTCTCTC
Caspase1 (NM_009807)	
Forward	GGAAGCAATTTATCAACTCAGTG
Reverse	GCCTTGTCCATAGCAGTAATG
Dicer-1 (NM_148948)	
Forward	TGTCATCTTGCGATTCTA
Reverse	TCTCTTCCAATTCCTCTG
DROSHA (NM_001130149)	
Forward	CTTCAACAGTTACCAGAAC
Reverse	CCTTTGGGAGTGAGTATG
AGO1 (NM_001317174)	
Forward	CCTGTGTATGATGGAAAGA
Reverse	CACTTGATGGAGACCTTAA
AGO2 (NM_153178)	
Forward	GGAGAACAATCAAACTACAG
Reverse	CAGATTCTTCCTTCCATCA
DGCR8 (NM_033324)	
Forward	CAGATAAGAAGGATGAGGAA
Reverse	GCTCCAAATTGTCAGTAAA
miR-155 (MIMAT0000165)	
Forward	UUAAUGCUAAUUGUGAUAGGGGUA
miR-203 (MIMAT0000236)	
Forward	GUGAAAUGUUUAGGACCACUAG
miR-29a (MIMAT0000535)	
Forward	UAGCACCAUCUGAAAUCGGUUA
miR-124-3p (MIMAT0000134)	
Forward	UAAGGCACGCGGUGAAUGCC

**Table 2:** Upregulated miRNAs in Zika virus (ZIKV)-infected neurons at 24 h and 48 h.

miRNA	Fold-change (24 h)	miRNA	Fold-change (48 h)
miR-155	18.29	miR-155	10.61
miR-3471	4.96	miR-203	4.61
miR-2145	2.33	miR-1902	2.95
miR-29a	2.09	miR-29b	2.1
miR-29b	2.05	miR-29a	2.08

**Table 3:** Downregulated miRNAs at 24 h.

miRNA	Fold-change	miRNA	Fold-change
miR-124-3p	−4.02	miR-144	−2.15
miR-M1-1	−2.97	miR-201	−2.15
miR-1892	−2.85	miR-764-5p	−2.15
miR-883a-3p	−2.7	miR-1895	−2.13
miR-879	−2.69	miR-871	−2.12
miR-669g	−2.65	miR-m108-1	−2.12
miR-654-3p	−2.62	miR-1928	−2.11
miR-339-3p	−2.52	miR-1941-5p	−2.11
miR-1960	−2.51	miR-759	−2.11
miR-207	−2.51	miR-1187	−2.1
miR-2861	−2.51	miR-666-3p	−2.1
miR-412	−2.48	miR-1966	−2.09
miR-741	−2.47	miR-1942	−2.08
miR-770-5p	−2.41	miR-1946a	−2.08
miR-673-5p	−2.38	miR-1967	−2.08
miR-1941-3p	−2.37	miR-m01-1	−2.07
miR-493	−2.33	miR-383	−2.06
miR-465a-3p	−2.31	miR-186	−2.05
miR-1956	−2.26	miR-323-5p	−2.05
miR-1194	−2.25	miR-M1-3	−2.05
miR-709	−2.25	miR-433	−2.04
miR-m107-1-5p	−2.25	miR-767	−2.04
miR-877	−2.23	miR-1188	−2.03
miR-331-5p	−2.22	miR-694	−2.03
miR-483	−2.22	miR-665	−2.02
miR-675-5p	−2.2	miR-2139	−2.02
miR-1957	−2.19	miR-883a-5p	−2.02
miR-M23-1-3p	−2.19	miR-1943	−2
miR-1898	−2.18	miR-346	−2
miR-1940	−2.17	miR-764-3p	−2
miR-1894-5p	−2.16	miR-710	−2

**Table 4:** Downregulated miRNAs at 48 h.

miRNA	Fold-change	miRNA	Fold-change
miR-124-3p	−6.68	miR-m21-1	−2.15
miR-883a-3p	−3.93	miR-335-3p	−2.14
miR-2137	−2.92	miR-1957	−2.12
miR-2133	−2.74	miR-764-5p	−2.11
miR-714	−2.56	miR-1194	−2.08
miR-669g	−2.54	miR-683	−2.08
miR-467g	−2.5	miR-509-5p	−2.07
miR-1188	−2.41	miR-463	−2.06
miR-879	−2.33	miR-741	−2.06
miR-760	−2.25	miR-761	−2.05
miR-298	−2.21	miR-710	−2.03
miR-m01-3	−2.21	miR-346	−2.01
miR-764-3p	−-2.2	miR-882	−2.01
miR-370	−2.18	miR-1898	−2.01
miR-1892	−2.16	miR-759	−2
miR-m01-2	−2.16	miR-433	−2
miR-133b	−2.15	miR-709	−2
miR-1894-5p	−2.15	miR-1956	−2
miR-666-3p	−2.15	miR-483	−2
miR-877	−2.15	miR-1941-5p	−2

**Table 5:** Top biological functions regulated by significantly modulated miRNAs.

Biological Process/Pathway	*p*-Value	Number of miRNAs
Cancer	4.02 × 10^−10^	18
Organismal Injury and Abnormalities	4.02 × 10^−10^	23
Reproductive System Disease	4.02 × 10^−10^	18
Immunological Disease	6.98 × 10^−10^	13
Inflammatory Disease	6.98 × 10^−10^	13
Inflammatory Response	6.98 × 10^−10^	11
Neurological Disease	6.98 × 10^−10^	10
Connective Tissue Disorders	1.46 × 10^−8^	10
Respiratory Disease	1.46 × 10^−8^	7
Nervous System Development and Function	7.20 × 10^−7^	6

**Table 6:** Upregulated mRNAs in ZIKV-infected neurons at 24 and 48 h.

mRNA	Fold-Change (24 h)	Fold-Change (48 h)	mRNA	Fold-Change (24 h)	Fold-Change (48 h)
Rsad2	26.44	244.24	Ccl2	3.91	14.32
Cxcl10	22.31	113.73	Ifna4	2.85	14.02
Ifit3	21.63	103.85	Nlrc5	3.42	13.39
Ifi44	18.36	103.12	Psmb10	3.13	12.78
Irf7	12.53	96.42	Stat2	5.55	12.37
Ifit1	24.52	75.06	H2-K1	2	12.34
Isg15	20.58	69.08	Cfb	2.04	12.04
Lcn2	27.07	68.88	Ifi35	4.05	11.3
Zbp1	13.57	55.1	Ddx58	3.84	10.56
Gbp5	12.51	44.43	Psmb9	2.4	10.03
Ifit2	2.53	41.91	Tap1	2.77	9.85
Ccl5	24.11	37.65	C3	3.29	9.22
Oas2	10.64	37.13	Casp1	2.76	7.63
Oasl1	5.15	34.55	Socs1	2.13	6.63
Usp18	9.7	32.04	H2-Ab1	2.21	5.82
Isg20	5.57	30.07	Cxcl1	3.49	5.5
H2-T23	3.61	28.33	Ifitm1	3.94	5.39
Ifih1	6.11	25.37	Ccl12	3.68	5.38
Ddx60	7.87	23.88	Pml	2	5.38
Stat1	6.92	21.99	Vcam1	2.23	5.24
Bst2	4.92	21.62	Myd88	2.38	4.87
Mx2	7.55	20.85	Cxcl9	2.83	4.7
Psmb8	4.31	20.39	Il6	2.07	3.63
Ccl7	2.71	19.98	Ptgs2	2.02	3.32
Cd274	4.02	19.34	Cxcl2	2.68	3.27
Cmpk2	4.01	18.95	Sbno2	3.05	2.86
Xaf1	8.02	18.06	Ccl4	2.52	2.84
Herc6	3.68	17.33	Lif	2.75	2.47
Ifnb1	2.97	16.75	Il13ra1	2.01	2.33
Tlr3	3.84	16.4	Runx1	2	2.15
Irgm2	6.29	15.13	Litaf	2	2.01

**Table 7:** Upregulated mRNAs in ZIKV-infected neurons at 48 h only.

mRNA	Fold-Change	mRNA	Fold-Change
Cxcl11	17.69	Slamf7	2.81
C2	9.88	Tnfrsf14	2.76
Tnfsf10	9.53	Fcgr4	2.6
Cxcl13	9.29	Mill2	2.59
Cd74	8.49	Ptprc	2.59
C1ra	7.96	H2-Dma	2.57
H2-D1	7.64	Nfkbia	2.52
H2-Aa	7.35	Tnfaip3	2.51
Tap2	6.53	Cxcl5	2.47
Ccl11	5.49	A2m	2.46
Fcgr1	5.32	Ifna1	2.38
H2-M3	5.25	Il3ra	2.36
Ccrl2	4.9	Ctss	2.34
Cd47	4.2	Ripk2	2.34
Tapbp	3.97	H2-DMb1	2.28
C4b	3.96	Lck	2.27
Ifna2	3.96	Cd80	2.26
Irf1	3.88	Cxcl16	2.19
Tlr2	3.87	Cybb	2.19
C1s1	3.56	Icosl	2.18
Lbp	3.44	Cfi	2.14
Il7	3.3	Irf2	2.1
Irf5	3.03	Nod1	2.02
Cd69	2.99	Atm	2
Serping1	2.91	Axl	2
Flt3l	2.87	H2-Eb1	2
Bid	2.85	Relb	2

**Table 8:** Downregulated mRNAs in ZIKV-infected neurons.

mRNA	Fold-Change (48 h)	mRNA	Fold-Change (48 h)
Cd36	−2	Cd207	−2.19
Elane	−2	Timd4	−2.22
Il17f	−2	Xcl1	−2.31
Ticam2	−2	Card9	−2.65
Pax5	−2.02	Sh2d1b1	−2.76
Il1rapl2	−2.15	Mpped1	−2.87
